# Diagnostic and Prognostic Roles of Blood-Based Immune Biomarkers in Non-Small Cell Lung Cancer: An Umbrella Review of Systematic Reviews and Meta-Analyses

**DOI:** 10.3390/life16071130

**Published:** 2026-07-07

**Authors:** Panpinhan Zhao, Rui Ling, Ruitong Li, Yiu-Wing Kam

**Affiliations:** Division of Natural and Applied Science, Duke Kunshan University, Suzhou 215316, China; panpinhan.zhao@dukekunshan.edu.cn (P.Z.); rui.ling@dukekunshan.edu.cn (R.L.); ruitong.li@dukekunshan.edu.cn (R.L.)

**Keywords:** non-small cell lung cancer, blood-based biomarkers, liquid biopsy, circulating tumor DNA, circulating tumor cells, non-coding RNAs, soluble immune biomarkers, umbrella review

## Abstract

Blood-based biomarkers have emerged as promising, minimally invasive tools for the diagnosis, prognostic stratification, and treatment monitoring of non-small cell lung cancer (NSCLC), including markers of tumor burden, tumor dissemination, immune signaling, and post-transcriptional regulation. However, evidence across biomarker classes remains fragmented. This study aimed to synthesize published evidence on major blood-based biomarkers relevant to diagnosis, prognosis, treatment stratification, and monitoring in NSCLC. PubMed was searched for systematic reviews and meta-analyses of blood-based biomarkers in NSCLC. Of 356 screened records, 82 underwent full-text review, and 57 systematic reviews/meta-analyses were included. Biomarkers were grouped into four categories: circulating tumor DNA (ctDNA), circulating tumor cells (CTCs), cytokines/soluble immune proteins, and non-coding RNAs (ncRNAs). Reported pooled effect estimates were extracted by biomarker class and evidence domain, and the methodological quality of included reviews was assessed using AMSTAR 2. Evidence was unevenly distributed across biomarker classes and evidence domains. Circulating ncRNAs were mainly represented in diagnostic and prognostic evidence; selected diagnostic ncRNAs, including miR-145, miR-25, and circRNAs, showed reported AUCs ranging from 0.83 to 0.85. ctDNA was represented across diagnostic, prognostic, treatment-stratification, and dynamic monitoring evidence, with ctDNA positivity associated with poorer survival or recurrence outcomes and ctDNA clearance or decline associated with improved outcomes. CTC evidence was primarily prognostic, with CTC positivity associated with worse overall survival and disease-free survival. Soluble immune biomarker evidence was also primarily prognostic, with elevated soluble PD-L1 and IL-6 associated with adverse survival outcomes and limited exploratory monitoring evidence for exosomal PD-L1. Overall, the evidence suggested distinct but complementary roles across biomarker classes, although direct head-to-head comparisons were lacking. Blood-based biomarkers show potential to support diagnosis, prognosis, and longitudinal monitoring in NSCLC, but their reported utility differs by biomarker class and clinical context. In the available review-level evidence, ncRNAs were mainly represented in diagnostic and prognostic evidence, while ctDNA was represented across diagnostic, prognostic, treatment-stratification, and dynamic monitoring evidence. CTCs were mainly represented in prognostic evidence, and soluble immune biomarkers were primarily represented in prognostic evidence, with limited exploratory evidence for dynamic monitoring. Further assay standardization, prospective validation, and direct comparative studies are needed before these biomarkers can be routinely integrated into clinical practice.

## 1. Introduction

Non-small cell lung cancer (NSCLC) is one of the most common and lethal malignancies worldwide, accounting for approximately 85% of all lung cancer cases [1,2]. Despite recent therapeutic advances, most patients are still diagnosed at advanced stages, and survival outcomes remain unsatisfactory [3]. NSCLC exhibits substantial biological and clinical heterogeneity, which contributes to variable treatment responses and survival outcomes, underscoring the urgent need for more effective early detection methods, reliable prognostic tools, and precision treatment strategies across all disease stages [4,5,6].

Blood-based biomarkers have emerged as promising, minimally invasive tools that can complement conventional tissue biopsy and radiographic assessment. Liquid biopsy technologies, including circulating tumor DNA (ctDNA), circulating tumor cells (CTCs), circulating non-coding RNAs (ncRNAs), and soluble immune-related proteins and cytokines, offer several advantages: they are minimally invasive, can be serially sampled for real-time monitoring, and may capture spatial and temporal tumor heterogeneity that is missed by single-site tissue biopsies [4,5,7]. These biomarkers reflect different biological dimensions (tumor burden, tumor dissemination, immune-inflammatory activity, and post-transcriptional regulation) and therefore may provide complementary information relevant to early diagnosis, prognosis assessment, treatment stratification, and longitudinal monitoring in NSCLC [7,8,9,10].

In current clinical practice, ctDNA-based molecular profiling is most established for advanced NSCLC when tissue is insufficient or when rapid genotyping is needed, though tissue biopsy remains essential when plasma testing is negative or when histological confirmation is required [11]. Beyond this setting, blood-based biomarkers are also needed for early detection, perioperative recurrence-risk assessment, treatment selection for both targeted therapy and immunotherapy, and dynamic monitoring of treatment response. For example, immune checkpoint inhibitors (ICIs) have become integral to advanced NSCLC management, and while tissue PD-L1 expression is used as a predictive biomarker for ICI benefit, its utility is limited by assay variability, threshold selection, and intratumoral heterogeneity [12,13,14,15]. In previously treated advanced or metastatic NSCLC, anti-PD-1/PD-L1 antibodies have shown clinical benefit compared with docetaxel in randomized trial evidence [13]. Only a subset of patients derives durable benefit from immunotherapy, and resistance remains a major challenge, highlighting the need for more reliable and reproducible biomarkers to guide patient stratification [16,17,18,19]. However, the need for blood-based biomarkers extends beyond the immunotherapy setting to include perioperative minimal residual disease (MRD) detection, adjuvant therapy decision-making, and monitoring of targeted therapy or chemotherapy responses.

Despite the growing volume of published research, evidence across different biomarker classes and clinical evidence domains remains fragmented. Individual systematic reviews and meta-analyses have typically focused on a single biomarker class or a single clinical application, making it difficult to compare the relative strengths and limitations of different biomarker types across diagnostic, prognostic, treatment-stratification, and monitoring contexts. To address this gap, we conducted an umbrella review of systematic reviews and meta-analyses to synthesize and compare the available review-level evidence for the four major blood-based biomarker classes (ctDNA, CTCs, ncRNAs, and soluble immune-related proteins) across the full spectrum of clinical applications in NSCLC, from early detection to treatment monitoring.

## 2. Methods

**Figure 1:**
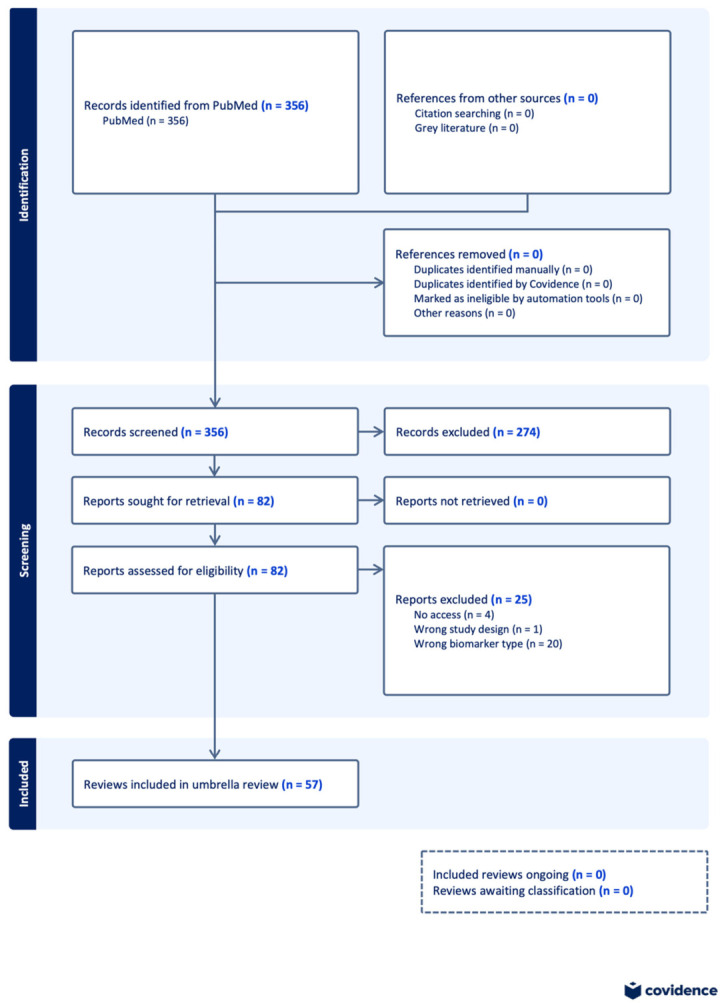
PRISMA flow diagram of review selection. A total of 356 records were imported from PubMed to Covidence. After title and abstract screening, 274 records were excluded, and 82 reports were sought for full-text retrieval. All 82 reports were successfully retrieved and assessed for eligibility. Of these, 25 reports were excluded for the following reasons: no access (n = 4), wrong study design (n = 1), and wrong biomarker type (n = 20). Ultimately, 57 systematic reviews and meta-analyses were included in the final umbrella review.

This study was designed as an umbrella review to evaluate the diagnostic, prognostic, treatment-stratification, and monitoring-related value of blood-based biomarkers in non-small cell lung cancer (NSCLC). This umbrella review was conducted and reported in accordance with the Preferred Reporting Items for Systematic Reviews and Meta-Analyses 2020 statement (PRISMA 2020) [20]. A completed PRISMA 2020 checklist is provided as Supplementary Table S1, and the study selection process is presented in the PRISMA flow diagram in Figure 1. The review protocol was registered in PROSPERO (CRD420261350843). The review focused on published systematic reviews and meta-analyses rather than individual primary studies, with the aim of synthesizing review-level evidence across multiple biomarker classes. An umbrella review design was selected because multiple systematic reviews and meta-analyses have already evaluated individual blood-based biomarker classes in NSCLC, whereas an overview across biomarker categories and evidence domains was needed to clarify broader evidence patterns, methodological limitations, and complementary clinical roles. Four major biomarker categories were examined: circulating tumor DNA (ctDNA), circulating tumor cells (CTCs), cytokines and soluble immune proteins, and non-coding RNAs (ncRNAs). These categories were selected because they capture complementary biological aspects of NSCLC.

A literature search was conducted on 25 May 2025 in PubMed using the search terms ((NSCLC) OR (non-small cell lung cancer)) AND (biomarkers). There were 23,124 results after searching. The “Systematic Review” and “Meta-Analysis” filters were then applied to focus the search on published systematic reviews and meta-analyses that examined blood-based biomarkers in NSCLC. After selection, 356 reviews were included in the screening stage. Reviews were considered eligible if they reported pooled quantitative estimates for diagnostic, prognostic, treatment-stratification, or monitoring-related outcomes and included biomarkers measured in blood-based specimens. Biomarkers of interest included ctDNA, CTCs, circulating proteins, cytokines, soluble immune markers, and ncRNAs. Because the aim of this umbrella review was to synthesize and compare published review-level quantitative estimates across biomarker classes and evidence domains, reviews without pooled quantitative estimates were not included in the main evidence synthesis. Reviews that did not focus on NSCLC, did not evaluate blood-based biomarkers, or did not provide pooled quantitative results were excluded.

Study selection was performed in two stages with Covidence. First, titles and abstracts were screened for relevance. Among 356 records identified after restriction to systematic reviews, 274 were excluded during initial screening, and 82 articles were retained for full-text assessment. In the second stage, full texts were reviewed against the predefined eligibility criteria, and 57 studies were included in the final synthesis. The study selection process is summarized in Figure 1. Three reviewers (PZ, RLing, and RLi) independently screened all retrieved records at two stages. Any disagreements were resolved through discussion.

Data extraction focused on pooled effect estimates reported in the included systematic reviews and meta-analyses. Extracted information included biomarker type, evidence domain, reported clinical endpoint, pooled hazard ratios (HRs), diagnostic performance measures such as sensitivity, specificity, and area under the receiver operating characteristic curve (AUC), and the source review or meta-analysis. Biomarkers were then grouped by biological category and evidence domain to allow cross-category comparison of diagnostic, prognostic, treatment-stratification, and monitoring-related roles. Because this study synthesized previously published pooled estimates, no individual patient-level data were reanalyzed. No additional meta-analysis or statistical re-pooling was performed in the present umbrella review. This decision was made because the included reviews differed substantially in biomarker definitions, assay platforms, specimen timing, clinical settings, outcome definitions, and statistical approaches. In addition, overlap of primary studies across included reviews was likely, which could have led to double-counting of evidence if new pooled estimates were generated across reviews. Therefore, the present study summarized and compared published pooled estimates descriptively rather than producing new pooled effect estimates. We did not conduct a formal quantitative assessment of primary-study overlap, such as the calculation of the corrected covered area, and the potential influence of overlap was therefore considered qualitatively when interpreting the evidence.

Quality assessment of the included systematic reviews and meta-analyses was conducted using AMSTAR 2 (A Measurement Tool to Assess Systematic Reviews 2) [21]. Two reviewers, PZ and RLing, independently evaluated each included review using a standardized assessment sheet. Disagreements were resolved through discussion and consensus. The AMSTAR 2 results were used to characterize the overall methodological quality of the included evidence base and to inform interpretation of the findings.

Quantitative synthesis focused on time-to-event outcomes reported as hazard ratios (HRs) in the included systematic reviews and meta-analyses. These published pooled HRs were extracted and compared across biomarker classes and clinical contexts. In general, HRs greater than 1 indicated poorer outcomes in the biomarker-positive or higher-risk group, whereas HRs less than 1 indicated more favorable outcomes in the comparison of interest, such as biomarker clearance or treatment benefit. For diagnostic biomarkers, pooled sensitivity, specificity, and area under the receiver operating characteristic curve (AUC) values reported in the source meta-analyses were summarized descriptively and compared visually across biomarkers. Consistent with the umbrella review design, these estimates were summarized descriptively and visually rather than statistically re-pooled.

All visualizations were generated in RStudio (2025.09.2+418) as part of the evidence-synthesis process. These visual summaries are provided as Supplementary Figures S1–S4. Forest plots were used to display pooled HRs for overall survival (OS), progression-free survival (PFS), recurrence-free survival (RFS), and disease-free survival (DFS), depending on the biomarker class and available evidence. Additional comparative plots were used to summarize diagnostic performance measures across biomarkers. The purpose of these visualizations was to facilitate interpretation of pooled evidence patterns across biomarker types rather than to replace the original published meta-analytic findings.

## 3. Results

The methodological quality of the included reviews varied according to the AMSTAR 2 assessment. Of the 57 included reviews, 18 were rated as moderate, 20 as low, and 19 as critically low in overall confidence. Thus, 39 of 57 reviews (68.4%) were rated as low or critically low. The most common critical weakness was the absence of a pre-established review protocol, followed by limitations in appropriate risk-of-bias assessment and consideration of risk of bias in interpretation.

### 3.1. Circulating Non-Coding RNAs (ncRNAs)

For circulating non-coding RNAs (ncRNAs), eligible review-level evidence was available for diagnostic accuracy and prognostic associations, but not for treatment-stratification or dynamic monitoring applications. Three diagnostic meta-analyses were included: Tao 2020 for miR-145, Li 2020 for miR-25, and Yu 2022 for circRNAs [22,23,24]. Three prognostic meta-analyses were also included: Pop-Bica 2020, Yuan 2018, and Xiong 2018 [25,26,27]. No eligible review-level evidence on treatment-stratification or dynamic monitoring roles of circulating ncRNAs was identified in the included meta-analyses.

Diagnostic accuracy for circulating ncRNAs was evaluated using pooled sensitivity, specificity, and area under the receiver operating characteristic curve (AUC), where available. miR-145 showed a pooled sensitivity of 0.78, specificity of 0.75, and AUC of 0.83 (95% CI: 0.80–0.86) (Supplementary Table S2). miR-25 showed a pooled sensitivity of 0.75, specificity of 0.81, and AUC of 0.85 (95% CI: 0.82–0.88) (Supplementary Table S2). CircRNAs showed a pooled sensitivity of 0.78, specificity of 0.76, and AUC of 0.84 (95% CI: 0.80–0.87) (Supplementary Table S2). Across these selected diagnostic meta-analyses, reported AUC values were above 0.80; however, the evidence was limited to specific ncRNA subtypes and should not be generalized to circulating ncRNAs as a whole.

Prognostic effects were evaluated primarily through pooled hazard ratios (HRs) for overall survival (OS). Among microRNAs, high circulating miR-21 expression was consistently associated with worse OS, with pooled HRs of 1.87 (95% CI: 1.41–2.47) and 1.96 (95% CI: 1.51–2.55) in two independent meta-analyses (Supplementary Table S2). Reduced circulating let-7 expression was also associated with worse survival outcomes, with a pooled HR of 2.61 (95% CI: 1.58–4.30) (Supplementary Table S2; Supplementary Figure S1A). Long non-coding RNAs (lncRNAs) showed different associations according to functional classification (Supplementary Table S2). Oncogenic lncRNAs were associated with increased mortality risk (HR = 1.18, 95% CI: 1.14–1.22), whereas tumor-suppressor lncRNAs were associated with improved survival outcomes (HR = 0.54, 95% CI: 0.44–0.66) (Supplementary Table S2). Overall, prognostic associations varied across ncRNA subtypes and functional categories, suggesting that prognostic interpretation should remain biomarker-specific rather than being applied to circulating ncRNAs as a single unified class (Table 1; Supplementary Table S2).

### 3.2. Circulating Tumor DNA (ctDNA)

For circulating tumor DNA (ctDNA), eligible review-level evidence was available for diagnostic accuracy, prognostic associations, and monitoring-related applications. Five meta-analyses met the inclusion criteria [28,29,30,31,32], covering ctDNA mutation profiling, ctDNA-based minimal residual disease (MRD), ctDNA methylation signatures, baseline or perioperative ctDNA status, and dynamic ctDNA changes in NSCLC.

Diagnostic evidence for ctDNA was available for mutation profiling, MRD detection, and methylation signatures. ctDNA mutation profiling showed a pooled sensitivity of 0.69 and specificity of 0.99 (Supplementary Table S2). ctDNA-based MRD detection showed a pooled sensitivity of 0.58 and specificity of 0.93 (Supplementary Table S2). ctDNA methylation signatures showed a pooled sensitivity of 0.62 and specificity of 0.90 (Supplementary Table S2). Overall, reported ctDNA-related diagnostic assays were characterized by relatively high specificity and more modest sensitivity across available meta-analyses, although the assays differed in target selection, clinical setting, and detection approach (Table 1; Supplementary Table S2).

Prognostic associations for ctDNA were reported across baseline, preoperative, and post-treatment ctDNA status for OS, RFS, and PFS. For OS, preoperative ctDNA positivity was associated with worse survival in Lu 2024 (HR = 2.77, 95% CI: 1.67–4.58) (Supplementary Table S2), while Guo 2023 reported adverse associations for both pre-treatment ctDNA positivity (HR = 3.82, 95% CI: 2.15–6.79) and post-treatment/MRD-positive ctDNA (HR = 4.73, 95% CI: 2.57–8.70) (Supplementary Table S2) [31,33]. Sun 2023 also reported worse OS among ctDNA-positive patients (HR = 2.33, 95% CI: 1.91–2.85) (Supplementary Table S2) [32]. For recurrence-related outcomes, preoperative ctDNA positivity was associated with worse RFS in both Lu 2024 (HR = 3.00, 95% CI: 2.26–3.98) and Guo 2023 (HR = 3.82, 95% CI: 2.74–5.32) (Supplementary Table S2) [31,33]. Post-treatment/MRD-positive ctDNA showed the largest reported association with RFS in Guo 2023 (HR = 8.32, 95% CI: 4.85–14.28) (Supplementary Table S2) [33]. In Sun 2023, ctDNA positivity was also associated with shorter PFS (HR = 2.34, 95% CI: 1.89–2.89) (Supplementary Table S2) [32]. Overall, adverse prognostic associations were reported across multiple ctDNA-related comparisons, although the clinical setting, timing of sampling, and outcome definition varied across meta-analyses (Table 1; Supplementary Table S2).

Monitoring-related evidence for ctDNA was identified for dynamic ctDNA change and treatment outcomes in ctDNA-positive patients. In Sun 2023, ctDNA clearance or decline during treatment was associated with improved OS (HR = 0.40, 95% CI: 0.27–0.60) and PFS (HR = 0.24, 95% CI: 0.19–0.31) (Supplementary Table S2) [32]. In Lu 2024, among patients with positive preoperative ctDNA, receipt of postoperative adjuvant therapy was associated with improved recurrence-free survival compared with no adjuvant treatment (HR = 0.39, 95% CI: 0.22–0.67) (Supplementary Table S2) [31]. These findings suggest that dynamic ctDNA change and ctDNA-positive clinical contexts may have monitoring or treatment-stratification relevance in available studies, but they should not be interpreted as definitive evidence of improved outcomes from ctDNA-based treatment selection without prospective validation (Table 1; Supplementary Table S2). Overall, ctDNA evidence showed three main patterns: high specificity but modest sensitivity in diagnostic settings, adverse prognostic associations for ctDNA positivity, and favorable survival associations for ctDNA clearance or decline during treatment (Table 1; Supplementary Table S2).

### 3.3. Circulating Tumor Cells (CTCs)

For circulating tumor cells (CTCs), eligible review-level evidence was limited to prognostic associations. One meta-analysis met the inclusion criteria for CTCs in NSCLC [34]. Reported pooled effect estimates were available for overall survival (OS) and disease-free survival (DFS) according to baseline, postoperative, and overall CTC status. No eligible review-level evidence on diagnostic accuracy, treatment-stratification, or dynamic monitoring roles of CTCs was identified in the included meta-analyses.

Prognostic evidence for CTCs was derived from one eligible meta-analysis (Table 1; Supplementary Table S2). Across reported comparisons, CTC positivity was associated with worse OS and DFS. For OS, baseline CTC positivity was associated with a pooled HR of 3.03 (95% CI: 2.32–3.98), postoperative CTC positivity with a pooled HR of 2.80 (95% CI: 1.95–4.02), and overall CTC positivity with a pooled HR of 2.95 (95% CI: 2.37–3.66) (Supplementary Table S2). For DFS, baseline CTC positivity was associated with a pooled HR of 2.95 (95% CI: 1.90–4.59), postoperative CTC positivity with a pooled HR of 2.73 (95% CI: 1.94–3.85), and overall CTC positivity with a pooled HR of 2.97 (95% CI: 2.08–4.22) (Supplementary Table S2). Overall, adverse prognostic associations were reported across baseline, postoperative, and overall CTC analyses, although the evidence was derived from a single eligible meta-analysis (Table 1; Supplementary Table S2).

### 3.4. Cytokine and Soluble Immune Biomarkers

For cytokine and soluble immune biomarkers, eligible review-level evidence was primarily prognostic, with limited exploratory evidence for dynamic monitoring. Three meta-analyses met the inclusion criteria: two focused on soluble or exosomal PD-L1-related biomarkers, and one evaluated circulating inflammatory cytokines and acute-phase immune proteins [35,36,37]. Reported endpoints included overall survival (OS) and progression-free survival (PFS), with HR values greater than 1 indicating worse outcomes in the higher-level or higher-risk biomarker group.

Prognostic associations for cytokine and soluble immune biomarkers were reported for soluble PD-L1 (sPD-L1), exosomal PD-L1 (exoPD-L1), and selected inflammatory biomarkers. Among the included soluble immune biomarkers, sPD-L1 was the most frequently evaluated and was associated with poorer survival outcomes across NSCLC-specific analyses. Elevated baseline sPD-L1 was associated with worse OS, with pooled HRs ranging from 1.81 to 2.32, and worse PFS, with pooled HRs ranging from 2.18 to 2.52 (Supplementary Table S2). In addition to PD-L1-related markers, inflammation-associated biomarkers showed variable prognostic associations. Elevated C-reactive protein (CRP, HR 1.30, 95% CI 1.09–1.54) and interleukin-6 (IL-6, HR 1.80, 95% CI 1.32–2.46) were associated with worse OS in NSCLC (Supplementary Table S2), whereas IL-8 (HR 1.93, 95% CI 0.98–3.81) and IL-10 (HR 1.62, 95% CI 0.73–3.57) did not show statistically significant associations based on the reported confidence intervals. Overall, multiple soluble immune and inflammatory biomarkers were associated with survival outcomes in available meta-analyses, but the strength and consistency of evidence varied across biomarkers and analytical contexts. No direct head-to-head comparisons between biomarker classes were conducted in the included meta-analyses, and comparative interpretations should therefore be made with caution.

Evidence for treatment-stratification or dynamic monitoring applications of cytokine and soluble immune biomarkers was limited and mainly exploratory. In Cui 2023, elevated baseline exosomal PD-L1 (exoPD-L1) was associated with worse PFS (HR = 4.44, 95% CI: 2.90–6.80), whereas a decrease in exoPD-L1 during treatment was associated with improved PFS (HR = 0.20, 95% CI: 0.06–0.64) (Supplementary Table S2). These findings suggest that dynamic exoPD-L1 changes may be relevant to treatment monitoring in available studies, but the evidence remains limited and requires prospective validation before clinical application. Overall, soluble immune biomarker evidence primarily supported survival-related risk stratification, with limited exploratory evidence for dynamic monitoring through exosomal PD-L1 changes (Table 1; Supplementary Table S2).

Across biomarker classes, the distribution of evidence domains was uneven. Diagnostic accuracy evidence was mainly available for circulating ncRNAs and ctDNA, whereas no eligible diagnostic accuracy evidence was identified for CTCs or cytokine/soluble immune biomarkers in the included reviews. Prognostic association was the most frequently represented evidence domain across biomarker classes, including selected ncRNAs, ctDNA positivity, CTC positivity, sPD-L1, CRP, and IL-6. In contrast, evidence for dynamic monitoring and treatment stratification was more limited and was concentrated mainly in ctDNA, with exploratory evidence for exosomal PD-L1. Overall, the current review-level evidence showed domain-specific clustering: ncRNAs were mainly represented in diagnosis and prognosis; ctDNA in diagnosis, prognosis, treatment stratification, and dynamic monitoring; CTCs in prognosis; and soluble immune biomarkers in prognosis with limited exploratory monitoring evidence (Table 1).

## 4. Discussion

This umbrella review synthesized evidence from 57 systematic reviews and meta-analyses to evaluate the diagnostic, prognostic, treatment-stratification, and monitoring-related relevance of blood-based biomarkers in NSCLC. Although the included biomarkers differed in their biological origins, they were considered within a unified blood-based biomarker framework because each may reflect clinically relevant aspects of tumor burden, tumor dissemination, systemic inflammation, immune escape, or tumor–host interaction [4,5,7,8,9,10,18,38,39,40]. The main finding of this review is that these biomarkers should not be interpreted as competing tools within a single hierarchy. Instead, the available review-level evidence suggests functional differentiation across biomarker classes and evidence domains: circulating ncRNAs were mainly represented in diagnostic and prognostic evidence; ctDNA in diagnostic, prognostic, treatment-stratification, and dynamic monitoring evidence; CTCs in prognostic evidence; and soluble immune biomarkers primarily in prognostic evidence, with limited exploratory evidence for dynamic monitoring (Table 1). These patterns should be interpreted as differences in the current evidence base rather than proof of comparative superiority, because direct head-to-head comparisons across biomarker classes were lacking. This functional differentiation is clinically important because the included biomarker classes do not necessarily address the same clinical question. Some markers are more closely aligned with disease discrimination, whereas others may better reflect residual disease, systemic dissemination, immune-inflammatory status, or longitudinal treatment response. Therefore, the interpretation of blood-based biomarkers in NSCLC should be organized around clinical use domains rather than around a single overall ranking of biomarker performance.

Among circulating ncRNAs, the main implication is that this biomarker class should not be treated as a single interchangeable category. Diagnostic meta-analyses reported potentially useful but biomarker-specific accuracy measures for selected circulating ncRNAs, including miR-145, miR-25, and circRNAs, while prognostic meta-analyses linked specific ncRNAs such as miR-21 and let-7 to overall survival (Table 1; Supplementary Table S2). These findings suggest that circulating ncRNAs may have diagnostic and prognostic relevance in available studies, but clinical translation will depend on validation of specific molecules, standardized assay strategies, and clearer threshold definitions rather than broad claims about ncRNAs as a whole [10,41]. This interpretation is biologically plausible because different ncRNAs participate in distinct post-transcriptional regulatory pathways related to tumor progression and immune modulation in NSCLC [9,10]. This also explains why ncRNA evidence should be interpreted at the level of individual molecules or functional categories. A circulating ncRNA with diagnostic discrimination may not necessarily have prognostic value, and an oncogenic ncRNA may carry an opposite clinical implication from a tumor-suppressive ncRNA. As a result, future studies should avoid pooling ncRNAs as a single homogeneous biomarker class unless molecular subtype, biological function, assay method, and clinical endpoint are clearly defined.

For ctDNA, the most consistent signal in this review related to recurrence-associated endpoints and longitudinal change. Preoperative or postoperative ctDNA positivity was associated with poorer survival outcomes, particularly recurrence-related outcomes, whereas ctDNA clearance or decline during treatment was associated with more favorable outcomes (Table 1; Supplementary Table S2). Mechanistically, persistent ctDNA may reflect minimal residual disease or ongoing tumor burden after therapy [33,42]. Clinically, this supports the potential relevance of ctDNA in postoperative surveillance and serial monitoring, where it may help identify patients at higher risk of occult residual disease or early relapse [33,42,43]. At the same time, the diagnostic pattern observed for ctDNA suggests that its clinical role may be more suitable for molecular confirmation, recurrence-risk assessment, and longitudinal follow-up than for standalone disease exclusion. The relatively modest sensitivity reported across ctDNA diagnostic assays indicates that negative ctDNA findings may not fully rule out disease, particularly in early-stage tumors or low-shedding contexts. However, the ctDNA literature remained heterogeneous in clinical settings, sampling time, assay targets, and positivity definitions, so ctDNA should be interpreted as a context-dependent biomarker rather than a single, uniform prognostic signal (Table 1).

The evidence for CTCs was primarily prognostic. Across baseline, postoperative, and overall analyses, CTC positivity was associated with worse OS and DFS (Table 1; Supplementary Table S2). Biologically, CTC detection may reflect systemic tumor dissemination and aggressive disease behavior, which supports its potential role in risk stratification rather than diagnosis or treatment selection at the current level of evidence [38,39,40]. This restricted evidence profile may also be related to methodological challenges in CTC detection. CTCs are rare in peripheral blood, and measured positivity can vary according to enrichment strategy, detection platform, epithelial or mesenchymal marker selection, and cut-off definition. These technical differences may limit cross-study comparability and may partly explain why the available review-level evidence is concentrated on prognostic endpoints rather than diagnostic accuracy or dynamic monitoring. However, because eligible review-level evidence was limited, further validation is needed before CTCs can be integrated into routine clinical decision-making.

For soluble immune biomarkers, elevated sPD-L1 was associated with poorer OS and PFS in NSCLC-specific analyses (Table 1; Supplementary Table S2). These findings suggest that blood-based immune markers may capture systemic host–tumor interactions not fully reflected by tissue-based assessment alone [8]. Mechanistically, sPD-L1 may reflect immune escape signaling, whereas inflammatory biomarkers such as CRP and IL-6 may reflect cancer-related inflammation and a more tumor-promoting systemic environment [7,8,37]. However, the interpretation of soluble immune biomarkers is limited by their relative lack of tumor specificity. Inflammatory markers may be influenced by infection, comorbid inflammatory disease, treatment-related inflammation, or other host factors, while soluble and exosomal PD-L1 measurements may vary by assay platform and cut-off definition. Therefore, these biomarkers may be more appropriately interpreted as contextual immune-inflammatory risk indicators rather than as standalone NSCLC-specific diagnostic or prognostic markers. Evidence for exoPD-L1 was exploratory, but treatment-related decreases were associated with improved PFS, suggesting that dynamic immune-related biomarkers may provide serial information on tumor–immune changes during treatment if validated prospectively [8,36,41].

Taken together, the findings support a complementary rather than substitutive view of blood-based biomarkers in NSCLC. The current evidence does not establish that any single biomarker class is superior to the others, nor does it support replacing tissue-based molecular testing, radiographic assessment, or established clinicopathological evaluation with a single blood-based marker [4,5,41]. Instead, blood-based biomarkers may have their greatest potential when used to address specific clinical questions and evidence domains: ncRNAs for diagnostic and prognostic assessment, ctDNA for diagnostic support, recurrence-risk assessment, treatment stratification, and longitudinal monitoring, CTCs for prognostic stratification, and soluble immune biomarkers for prognostic and immune-inflammatory outcome assessment, with exploratory monitoring evidence for exosomal PD-L1 (Table 1). Future studies should prioritize prospective validation in clinically defined NSCLC populations, standardized assay methods, harmonized thresholds, predefined sampling time points, and direct comparative or multimodal biomarker designs [4,5,41]. Importantly, multimodal biomarker development should be clinically question-driven rather than based on indiscriminate marker accumulation. For example, integrated panels could test whether ctDNA-based residual disease assessment, CTC-based dissemination risk, ncRNA-based diagnostic discrimination, and soluble immune-inflammatory markers provide non-overlapping information when evaluated alongside imaging, tissue genotyping, and clinicopathological variables. Such studies are needed to determine whether integrated blood-based biomarker strategies provide incremental value beyond current clinical, radiographic, tissue-based, and molecular assessments.

Beyond statistical performance and reported effect estimates, the clinical translation of blood-based biomarkers in NSCLC faces several practical barriers that were not systematically quantified in the included reviews. Assay standardization remains a major challenge across all biomarker classes: ctDNA platforms vary in panel design, sequencing depth, and bioinformatic algorithms; CTC detection is affected by enrichment methods, marker selection, and low abundance; and ncRNA and soluble protein assays lack harmonized reference materials and standardized thresholds [4,5,41]. These methodological differences limit cross-study comparability and complicate the establishment of clinically actionable cut-offs. Furthermore, integrating these biomarkers into routine practice will require clear algorithms that define how blood-based results should be interpreted alongside tissue genotyping, imaging, and clinicopathological variables—particularly when plasma ctDNA testing is negative or when tissue and blood findings are discordant [11]. Accessibility and cost-effectiveness, while critically important for real-world implementation, were not formally assessed in the meta-analyses included in this umbrella review; the available evidence does not yet permit robust health economic comparisons across biomarker classes. Moving forward, prospective validation in multicenter, clinically defined NSCLC cohorts is essential, alongside studies that evaluate whether integrated multimarker strategies—combining ctDNA, CTCs, ncRNAs, and soluble immune markers—provide incremental clinical value beyond current standard-of-care assessments. Such studies, ideally paired with health economic analyses, will be necessary to determine whether these biomarkers can be feasibly and cost-effectively incorporated into diagnostic and monitoring algorithms in routine oncology practice [41].

## 5. Limitations

Several limitations should be acknowledged. First, this umbrella review was based on published systematic reviews and meta-analyses rather than primary-study or patient-level data, which limited the ability to harmonize endpoints, biomarker thresholds, assay methods, and patient-level covariates. Second, the literature search was conducted only in PubMed and used a relatively broad search strategy based on NSCLC, non-small cell lung cancer, and biomarkers, followed by systematic review and meta-analysis filters. Although PubMed provides broad coverage of biomedical literature and allowed identification of a substantial number of eligible systematic reviews and meta-analyses, the absence of additional databases such as Embase, Web of Science, Scopus, or the Cochrane Library, as well as the use of a relatively simple search strategy, may have reduced the comprehensiveness of the evidence base and increased the possibility that relevant reviews were missed. Third, substantial heterogeneity existed across the included reviews in assay platforms, specimen timing, target selection, cut-off definitions, treatment context, and outcome definitions, particularly for ctDNA and soluble immune biomarkers. This lack of standardized assays limited comparability across studies and remains a major barrier to clinical translation [41]. Fourth, because the main synthesis was restricted to reviews with pooled quantitative estimates, systematic reviews without meta-analysis or without extractable pooled estimates were not included, which may have excluded relevant qualitative evidence. Some biomarker classes were represented by relatively few eligible meta-analyses, especially for diagnostic, treatment-stratification, and dynamic monitoring applications, which may limit generalizability. In addition, the apparent alignment between biomarker classes and clinical applications reflected the domains for which pooled estimates were available in the included reviews. Therefore, the finding that certain biomarker classes were mainly represented in particular clinical domains should not be interpreted as evidence that they lack utility in other contexts. Rather, it reflects the current distribution of published review-level evidence and underscores the need for broader comparative studies across clinical applications. Fifth, overlap of primary studies across included reviews was likely and represents a major methodological limitation. Repeated inclusion of the same underlying primary studies may have overestimated the apparent consistency of evidence across reviews. This concern also limited the appropriateness of generating additional pooled estimates in the present umbrella review. In addition, because this umbrella review synthesized published meta-analyses rather than reanalyzing primary studies, we did not perform new publication-bias assessments such as funnel plots or Egger tests. Publication bias within the included reviews or their underlying primary studies may therefore have influenced the reported pooled estimates. Finally, the methodological quality of the included reviews was variable. In the AMSTAR 2 assessment, 39 of 57 reviews (68.4%) were rated as low or critically low, with frequent weaknesses including absence of a pre-established protocol, limitations in risk-of-bias assessment, and insufficient consideration of bias when interpreting results [21]. AMSTAR 2 evaluates the methodological quality of systematic reviews, but it does not provide outcome-level certainty ratings for individual biomarker–outcome associations. We did not perform a separate GRADE or equivalent outcome-level certainty assessment; therefore, the strength of evidence for specific biomarker applications should be interpreted cautiously. Overall, the evidence base should be considered hypothesis-generating rather than practice-changing at this time.

## 6. Conclusions

This umbrella review synthesized evidence from 57 systematic reviews and meta-analyses on blood-based biomarkers in non-small cell lung cancer (NSCLC). The available review-level evidence suggests functional differentiation across biomarker classes and evidence domains rather than definitive superiority of any single biomarker type. Circulating ncRNAs were mainly represented in diagnostic and prognostic evidence; ctDNA in diagnostic, prognostic, treatment-stratification, and dynamic monitoring evidence; CTCs in prognostic evidence; and soluble immune biomarkers primarily in prognostic evidence, with limited exploratory evidence for dynamic monitoring [22,23,24,25,26,27,28,29,30,31,32,34,35,36,37]. These findings suggest that blood-based biomarkers may provide complementary information across different clinical questions in NSCLC, including diagnosis, prognosis, recurrence-risk assessment, treatment stratification, and longitudinal monitoring.

Clinically, blood-based biomarkers offer potential advantages because they are minimally invasive and can support repeated assessment over time [4,5,41]. However, their broader implementation remains constrained by heterogeneity in assay platforms, sampling strategies, cut-off definitions, and clinical contexts [28,29,30,31,32,41]. The current evidence does not support replacing existing tissue-based or radiographic assessment with any single blood-based biomarker. Instead, future research should prioritize prospective validation, standardized testing protocols, harmonized thresholds, and direct comparative or multimodal studies to determine whether integrated biomarker approaches provide incremental clinical value [41].

In conclusion, blood-based biomarkers represent a promising but still evolving component of precision management in NSCLC. Their current value lies in their potential to complement existing diagnostic, prognostic, and monitoring strategies rather than to serve as stand-alone clinical tools. Further methodological standardization and high-quality prospective evidence are needed before routine clinical integration [41].

## Figures and Tables

**Table 1:** Summary of review-level evidence for blood-based biomarkers in NSCLC.

Biomarker Class	Evidence Domains	Clinical Setting/Treatment Context	Representative Findings and Key Interpretation
Circulating non-coding RNAs (ncRNAs)	Diagnostic accuracy; prognostic association	Diagnostic discrimination and survival risk stratification; eligible treatment-specific and dynamic monitoring evidence was not identified among the included meta-analyses	Selected diagnostic ncRNAs showed moderate diagnostic performance, with reported AUCs of 0.83 for miR-145, 0.85 for miR-25, and 0.84 for circRNAs. Prognostic evidence linked miR-21, let-7, and selected lncRNA categories with OS outcomes. Interpretation should remain biomarker-specific because ncRNAs differ by molecule type, biological function, assay method, and threshold definition. Full estimates are provided in Supplementary Table S2.
Circulating tumor DNA (ctDNA)	Diagnostic accuracy; prognostic association; treatment-stratification evidence; dynamic monitoring	Advanced NSCLC molecular detection; localized or perioperative MRD/recurrence-risk assessment; postoperative adjuvant therapy context; longitudinal treatment monitoring	ctDNA-related diagnostic assays generally showed high specificity but modest sensitivity. ctDNA positivity before or after treatment was associated with poorer survival or recurrence outcomes, while ctDNA clearance or decline during treatment was associated with improved OS and PFS. Adjuvant therapy in ctDNA-positive patients was also associated with improved RFS in one included meta-analysis. These findings support potential roles in recurrence-risk assessment and longitudinal monitoring, but prospective validation is needed before ctDNA-guided treatment selection can be considered clinically established. Full estimates are provided in Supplementary Table S2.
Circulating tumor cells (CTCs)	Prognostic association	Baseline, postoperative, and overall prognostic stratification; no eligible diagnostic, treatment-stratification, or dynamic monitoring evidence was identified in the included meta-analyses	CTC positivity was consistently associated with worse OS and DFS across baseline, postoperative, and overall analyses in one eligible meta-analysis. The current review-level evidence therefore supports CTCs mainly as prognostic markers rather than diagnostic or treatment-selection tools. However, evidence was limited to a single eligible meta-analysis, and CTC detection remains affected by platform, enrichment method, marker selection, and cut-off heterogeneity. Full estimates are provided in Supplementary Table S2.
Cytokines and soluble immune biomarkers	Prognostic association; limited exploratory dynamic monitoring	Immune-related survival risk stratification, mainly in NSCLC-specific and ICI-treated contexts for PD-L1-related markers; exploratory treatment monitoring for exosomal PD-L1	Elevated soluble PD-L1 was associated with worse OS and PFS, while CRP and IL-6 were associated with worse OS in available NSCLC-specific analyses. Dynamic decreases in exosomal PD-L1 were associated with improved PFS in exploratory evidence. These biomarkers may reflect systemic immune-inflammatory status and tumor–host interaction, but interpretation is limited by low tumor specificity, assay heterogeneity, variable cut-offs, and limited prospective validation. Full estimates are provided in Supplementary Table S2.

Note: This table summarizes the main review-level evidence patterns by biomarker class. Detailed source-level estimates, including sensitivity, specificity, AUC, HRs, confidence intervals, and individual biomarker–outcome pairs, are provided in Supplementary Table S2. Evidence domains reflect the availability of pooled estimates in the included systematic reviews and meta-analyses and should not be interpreted as proof that a biomarker class lacks utility in other clinical contexts. Treatment context was summarized when reported in the included reviews; however, the number of ICI-treated or chemotherapy-treated trials was not consistently extractable across all source meta-analyses.

## Data Availability

This study is an umbrella review based on previously published systematic reviews and meta-analyses. All extracted review-level data and materials used for evidence synthesis are available from the corresponding author (YWK) upon reasonable request.

## Supplementary Materials

The following supporting information can be downloaded at: https://www.mdpi.com/article/10.3390/life16071130/s1, Figure S1: Reported diagnostic and prognostic effect estimates for circulating non-coding RNAs in NSCLC; Figure S2: Reported diagnostic, prognostic, and monitoring-related effect estimates for ctDNA-related biomarkers in NSCLC; Figure S3: Reported prognostic effect estimates for circulating tumor cells in NSCLC; Figure S4: Reported prognostic and monitoring-related effect estimates for blood-based cytokines and soluble immune biomarkers in NSCLC; Table S1: PRISMA 2020 Checklist; Table S2: Published diagnostic, prognostic, treatment-stratification, and monitoring-related effect estimates for blood-based biomarkers in NSCLC.
