# Supplementary material for: Diagnostic and Prognostic Roles of Blood-Based Immune Biomarkers in Non-Small Cell Lung Cancer: An Umbrella Review of Systematic Reviews and Meta-Analyses
Source: Life (Basel). 2026 Jul 7;16(7):1130. doi: 10.3390/life16071130 (PMC13412772; doi:10.3390/life16071130)
Supplement: Supplementary file 1 [file life-16-01130-s001.zip › Zhaoa_Ling_Li_Kam_manuscript final_July_6.pdf]

(A)

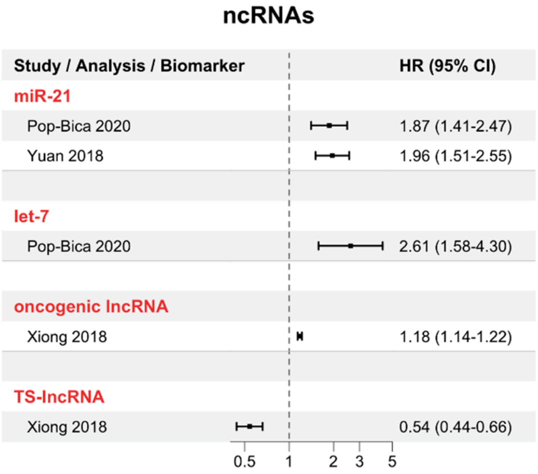

(B)

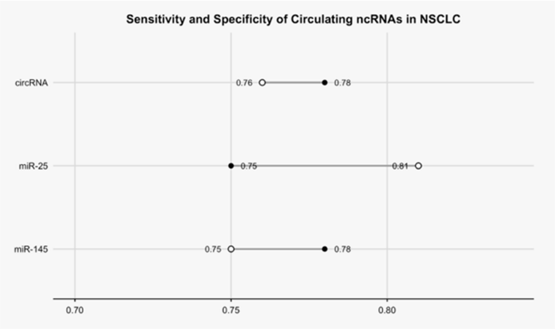

(C)

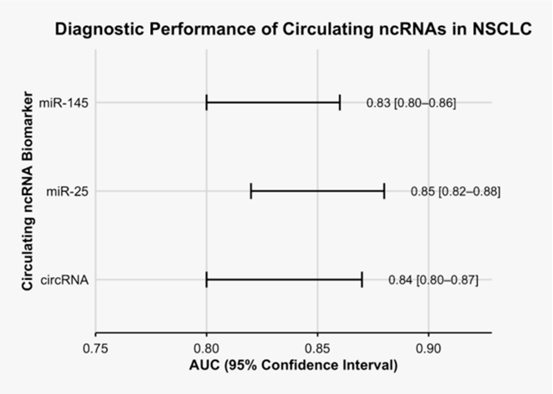

Supplementary Figure S1. Reported diagnostic and prognostic effect estimates for circulating non-coding RNAs in NSCLC. (A) Forest-style plot of published pooled hazard ratios (HRs) and 95% confidence intervals (CIs) for overall survival (OS) associated with selected circulating ncRNA biomarkers, including miR-21, let-7, oncogenic long non-coding RNAs (lncRNAs), and tumor-suppressor lncRNAs. HRs greater than 1 indicate worse survival in the higher-expression or biomarker-positive

group, whereas HRs less than 1 indicate more favorable survival in the comparison of interest. (B) Comparison of published pooled sensitivity and specificity values for selected diagnostic ncRNA biomarkers, including miR-145, miR-25, and circRNAs. In panel B, solid circles represent sensitivity and open circles represent specificity. (C) Comparison of published pooled area under the receiver operating characteristic curve (AUC) values with 95% CIs for the same diagnostic ncRNA biomarkers. All estimates were extracted from published systematic reviews and meta-analyses and are displayed without additional statistical pooling in the present umbrella review.

(A)

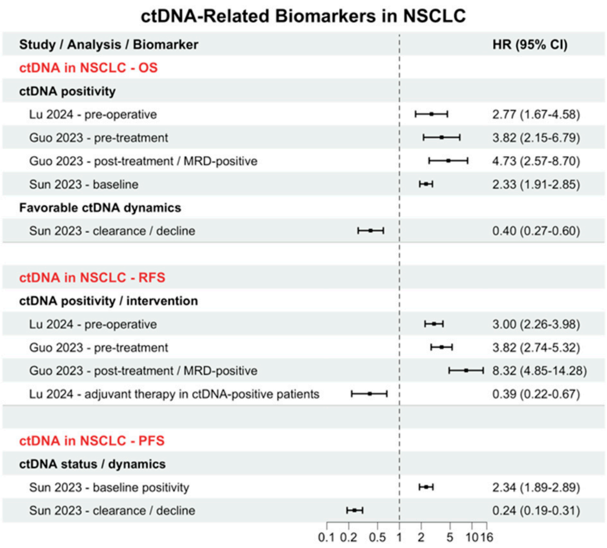

(B)

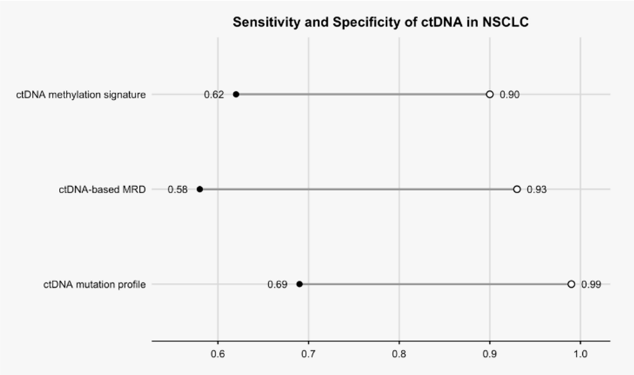

Supplementary Figure S2. Reported diagnostic, prognostic, and monitoring-related effect estimates for ctDNA-related biomarkers in NSCLC. (A) Forest-style plot of published pooled hazard ratios (HRs) and 95% confidence intervals (CIs) for ctDNA-related biomarkers across overall survival (OS), recurrence-free survival (RFS), and progression-free survival (PFS), including ctDNA positivity, post-treatment/MRD-positive status, adjuvant therapy in ctDNA-positive patients, and favorable ctDNA dynamics. HRs greater than 1 indicate worse outcomes in the ctDNA-positive or higher-risk group, whereas HRs less than 1 indicate more favorable outcomes in the comparison of interest, such as ctDNA clearance/decline or adjuvant treatment benefit. (B) Comparison of published pooled sensitivity and specificity values for selected ctDNA-related diagnostic assays, including ctDNA mutation profiling, ctDNA-based

MRD detection, and ctDNA methylation signatures. In panel B, solid circles represent sensitivity and open circles represent specificity. All estimates were extracted from published systematic reviews and meta-analyses and are displayed without additional statistical pooling in the present umbrella review.

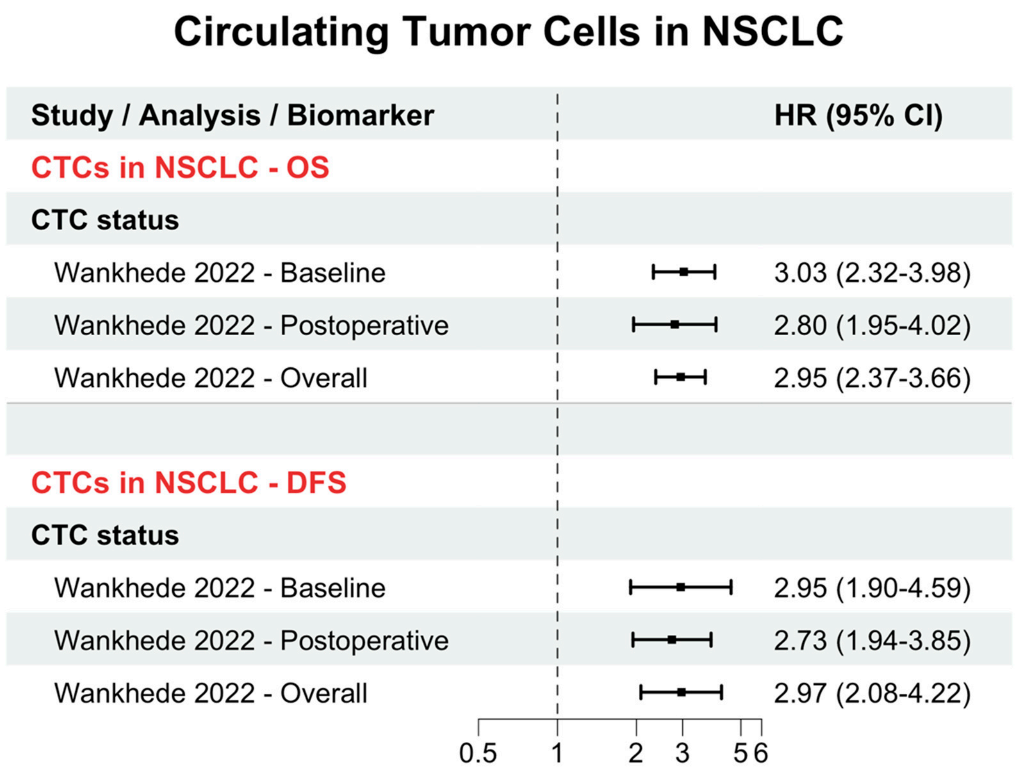

Supplementary Figure S3. Reported prognostic effect estimates for circulating tumor cells in NSCLC. Forest-style plot of published pooled hazard ratios (HRs) and 95% confidence intervals (CIs) for overall survival (OS) and disease-free survival (DFS) according to baseline, postoperative, and overall CTC status. HRs greater than 1 indicate worse outcomes in the CTC-positive group. All estimates were extracted from published systematic reviews and meta-analyses and are displayed without additional statistical pooling in the present umbrella review.

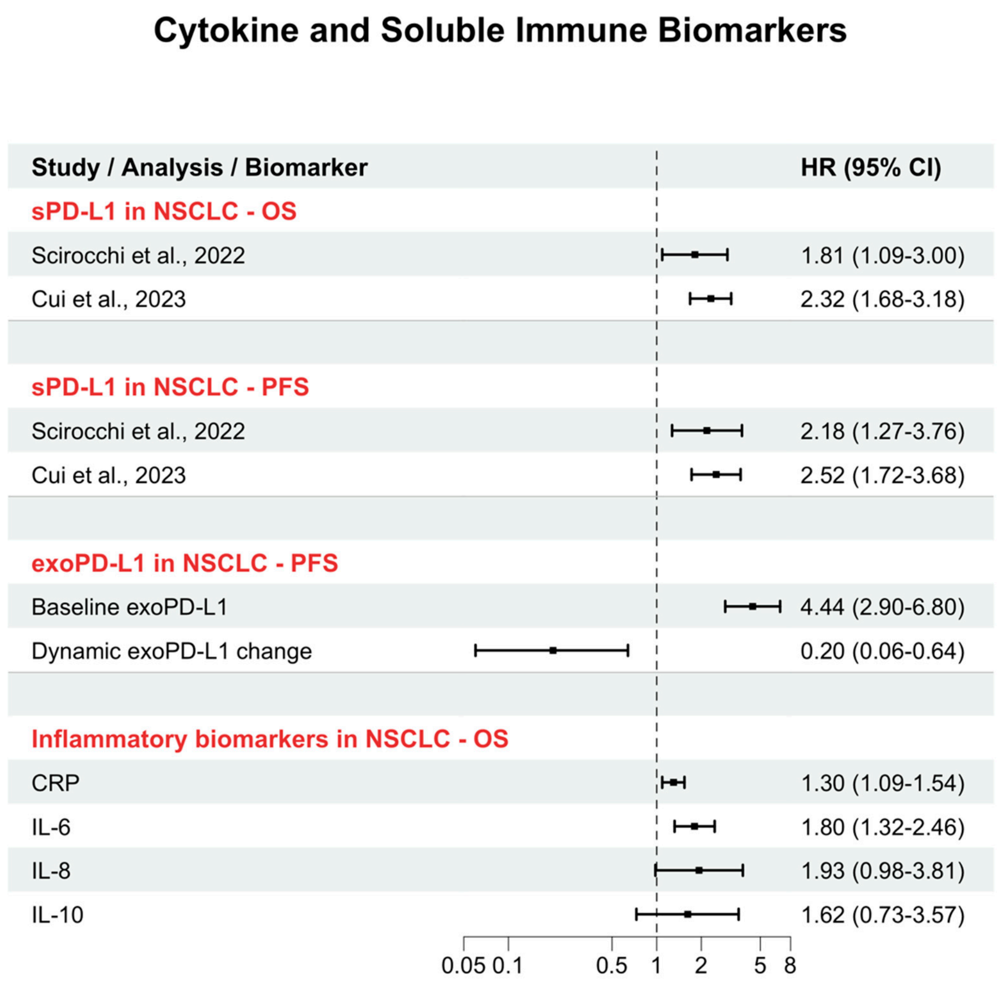

Supplementary Figure S4. Reported prognostic and monitoring-related effect estimates for blood-based cytokines and soluble immune biomarkers in NSCLC. Forest-style plot of published pooled hazard ratios (HRs) and 95% confidence intervals (CIs) for overall survival (OS) and progression-free survival (PFS) associated with soluble PD-L1 (sPD-L1), exosomal PD-L1 (exoPD-L1), and circulating inflammatory biomarkers. HRs greater than 1 indicate worse outcomes in the higher-level or higher-risk group, whereas HRs less than 1 indicate more favorable outcomes in the comparison of interest, including dynamic decreases in exoPD-L1. Because the estimates include both NSCLC-specific prognostic analyses and exploratory dynamic monitoring analyses, direct comparison should be interpreted cautiously. All estimates

were extracted from published systematic reviews and meta-analyses and displayed without additional statistical pooling in the present umbrella review.

Supplementary Table S2. Published diagnostic, prognostic, treatment-stratification, and monitoring-related effect estimates for blood-based biomarkers in NSCLC.

Biomarker class	Evidence domain	Source	Biomarker/analysis	Reported Estimate	Potential clinical decision context	Interpretation/gap
ncRNAs	Diagnostic accuracy	Tao 2020 [22]	miR-145	Sensitivity: 0.78 (95% CI: 0.71–0.83)	Adjunctive diagnostic discrimination for NSCLC	Moderate diagnostic performance, but evidence is biomarker-specific and requires assay and threshold standardization.
ncRNAs	Diagnostic accuracy	Tao 2020 [22]	miR-145	Specificity: 0.75 (95% CI: 0.67–0.82)	Adjunctive diagnostic discrimination for NSCLC	Moderate diagnostic performance, but evidence is biomarker-specific and requires assay and threshold standardization.
ncRNAs	Diagnostic accuracy	Tao 2020 [22]	miR-145	AUC: 0.83 (95% CI: 0.80–0.86)	Adjunctive diagnostic discrimination for NSCLC	Moderate diagnostic performance, but evidence is biomarker-specific and requires assay and threshold standardization.
ncRNAs	Diagnostic accuracy	Li 2020 [23]	miR-25	Sensitivity: 0.75 (95% CI: 0.69–0.80)	Adjunctive diagnostic discrimination for NSCLC	Moderate diagnostic performance, but evidence is biomarker-specific and requires assay and threshold standardization.

Biomarker class	Evidence domain	Source	Biomarker/analysis	Reported Estimate	Potential clinical decision context	Interpretation/gap
ncRNAs	Diagnostic accuracy	Li 2020 [23]	miR-25	Specificity: 0.81 (95% CI: 0.76–0.86)	Adjunctive diagnostic discrimination for NSCLC	Moderate diagnostic performance, but evidence is biomarker-specific and requires assay and threshold standardization.
ncRNAs	Diagnostic accuracy	Li 2020 [23]	miR-25	AUC: 0.85 (95% CI: 0.82–0.88)	Adjunctive diagnostic discrimination for NSCLC	Moderate diagnostic performance, but evidence is biomarker-specific and requires assay and threshold standardization.
ncRNAs	Diagnostic accuracy	Yu 2022 [24]	circRNAs	Sensitivity: 0.78 (95% CI: 0.71–0.83)	Adjunctive diagnostic discrimination for NSCLC	Moderate diagnostic performance, but evidence is biomarker-specific and requires assay and threshold standardization.
ncRNAs	Diagnostic accuracy	Yu 2022 [24]	circRNAs	Specificity: 0.76 (95% CI: 0.70–0.82)	Adjunctive diagnostic discrimination for NSCLC	Moderate diagnostic performance, but evidence is biomarker-specific and requires assay and threshold standardization.
ncRNAs	Diagnostic accuracy	Yu 2022 [24]	circRNAs	AUC: 0.84 (95% CI: 0.80–0.87)	Adjunctive diagnostic discrimination for NSCLC	Moderate diagnostic performance, but evidence is biomarker-specific and requires assay and threshold standardization.

Biomarker class	Evidence domain	Source	Biomarker/analysis	Reported Estimate	Potential clinical decision context	Interpretation/gap
ncRNAs	Prognostic association	Pop-Bica 2020 [25]	miR-21	OS HR: 1.87 (95% CI: 1.41–2.47)	Survival risk stratification	Associated with OS, but prognostic interpretation should remain biomarker-specific rather than generalized to all circulating ncRNAs.
ncRNAs	Prognostic association	Yuan 2018 [26]	miR-21	OS HR: 1.96 (95% CI: 1.51–2.55)	Survival risk stratification	Associated with OS, but prognostic interpretation should remain biomarker-specific rather than generalized to all circulating ncRNAs.
ncRNAs	Prognostic association	Pop-Bica 2020 [25]	let-7	OS HR: 2.61 (95% CI: 1.58–4.30)	Survival risk stratification	Associated with OS, but prognostic interpretation should remain biomarker-specific rather than generalized to all circulating ncRNAs.

Biomarker class	Evidence domain	Source	Biomarker/analysis	Reported Estimate	Potential clinical decision context	Interpretation/gap
ncRNAs	Prognostic association	Xiong 2018 [27]	Oncogenic lncRNAs	OS HR: 1.18 (95% CI: 1.14–1.22)	Survival risk stratification	Higher oncogenic lncRNA expression was associated with worse OS; functional classification and assay thresholds require further validation.
ncRNAs	Prognostic association	Xiong 2018 [27]	Tumor-suppressor lncRNAs	OS HR: 0.54 (95% CI: 0.44–0.66)	Survival risk stratification	Tumor-suppressor lncRNAs showed a protective association, but interpretation depends on lncRNA subtype and expression definition.
ctDNA	Diagnostic accuracy	Chen 2024 [28]	ctDNA mutation profile	Sensitivity: 0.69	Molecular detection and diagnostic support in advanced NSCLC	High specificity but modest sensitivity; clinical utility depends on assay target, timing, and disease setting.
ctDNA	Diagnostic accuracy	Chen 2024 [28]	ctDNA mutation profile	Specificity: 0.99	Molecular detection and diagnostic support in advanced NSCLC	High specificity but modest sensitivity; clinical utility depends on assay target, timing, and disease setting.

Biomarker class	Evidence domain	Source	Biomarker/analysis	Reported Estimate	Potential clinical decision context	Interpretation/gap
ctDNA	Diagnostic accuracy	Guo 2023 [29]	ctDNA-based MRD	Sensitivity: 0.58	MRD detection and recurrence-risk evaluation	High specificity but modest sensitivity; clinical utility depends on assay target, timing, and disease setting.
ctDNA	Diagnostic accuracy	Guo 2023 [29]	ctDNA-based MRD	Specificity: 0.93	MRD detection and recurrence-risk evaluation	High specificity but modest sensitivity; clinical utility depends on assay target, timing, and disease setting.
ctDNA	Diagnostic accuracy	Maffeo 2024 [30]	ctDNA methylation signature	Sensitivity: 0.62	Blood-based diagnostic discrimination or early detection support	High specificity but modest sensitivity; clinical utility depends on assay target, timing, and disease setting.
ctDNA	Diagnostic accuracy	Maffeo 2024 [30]	ctDNA methylation signature	Specificity: 0.90	Blood-based diagnostic discrimination or early detection support	High specificity but modest sensitivity; clinical utility depends on assay target, timing, and disease setting.

Biomarker class	Evidence domain	Source	Biomarker/analysis	Reported Estimate	Potential clinical decision context	Interpretation/gap
ctDNA	Prognostic association	Lu 2024 [31]	Preoperative ctDNA positivity	OS HR: 2.77 (95% CI: 1.67–4.58)	Recurrence and survival risk stratification	ctDNA positivity was associated with poorer survival or recurrence outcomes, but sampling time and positivity definitions varied across studies.
ctDNA	Prognostic association	Lu 2024 [31]	Preoperative ctDNA positivity	RFS HR: 3.00 (95% CI: 2.26–3.98)	Recurrence and survival risk stratification	ctDNA positivity was associated with poorer survival or recurrence outcomes, but sampling time and positivity definitions varied across studies.
ctDNA	Prognostic association	Guo 2023 [29]	Pre-treatment ctDNA positivity	OS HR: 3.82 (95% CI: 2.15–6.79)	Recurrence and survival risk stratification	ctDNA positivity was associated with poorer survival or recurrence outcomes, but sampling time and positivity definitions varied across studies.

Biomarker class	Evidence domain	Source	Biomarker/analysis	Reported Estimate	Potential clinical decision context	Interpretation/gap
ctDNA	Prognostic association	Guo 2023 [29]	Pre-treatment ctDNA positivity	RFS HR: 3.82 (95% CI: 2.74–5.32)	Recurrence and survival risk stratification	ctDNA positivity was associated with poorer survival or recurrence outcomes, but sampling time and positivity definitions varied across studies.
ctDNA	Prognostic association	Guo 2023 [29]	Post-treatment / MRD-positive ctDNA	OS HR: 4.73 (95% CI: 2.57–8.70)	Recurrence and survival risk stratification	ctDNA positivity was associated with poorer survival or recurrence outcomes, but sampling time and positivity definitions varied across studies.
ctDNA	Prognostic association	Guo 2023 [29]	Post-treatment / MRD-positive ctDNA	RFS HR: 8.32 (95% CI: 4.85–14.28)	Recurrence and survival risk stratification	ctDNA positivity was associated with poorer survival or recurrence outcomes, but sampling time and positivity definitions varied across studies.

Biomarker class	Evidence domain	Source	Biomarker/analysis	Reported Estimate	Potential clinical decision context	Interpretation/gap
ctDNA	Prognostic association	Sun 2023 [32]	Baseline ctDNA positivity	OS HR: 2.33 (95% CI: 1.91–2.85)	Recurrence and survival risk stratification	ctDNA positivity was associated with poorer survival or recurrence outcomes, but sampling time and positivity definitions varied across studies.
ctDNA	Prognostic association	Sun 2023 [32]	Baseline ctDNA positivity	PFS HR: 2.34 (95% CI: 1.89–2.89)	Recurrence and survival risk stratification	ctDNA positivity was associated with poorer survival or recurrence outcomes, but sampling time and positivity definitions varied across studies.
ctDNA	Treatment-stratification evidence	Lu 2024 [31]	Adjuvant therapy in ctDNA-positive patients	RFS HR: 0.39 (95% CI: 0.22–0.67)	Identifying ctDNA-positive patients who may benefit from postoperative adjuvant therapy	Suggests potential treatment-stratification value in ctDNA-positive patients, but prospective validation is needed before clinical implementation.

Biomarker class	Evidence domain	Source	Biomarker/analysis	Reported Estimate	Potential clinical decision context	Interpretation/gap
ctDNA	Dynamic monitoring	Sun 2023 [32]	ctDNA clearance / decline during treatment	OS HR: 0.40 (95% CI: 0.27–0.60)	Longitudinal treatment response monitoring	ctDNA clearance or decline was associated with improved outcomes, supporting monitoring relevance, but assay timing and response definitions require standardization.
ctDNA	Dynamic monitoring	Sun 2023 [32]	ctDNA clearance / decline during treatment	PFS HR: 0.24 (95% CI: 0.19–0.31)	Longitudinal treatment response monitoring	ctDNA clearance or decline was associated with improved outcomes, supporting monitoring relevance, but assay timing and response definitions require standardization.
CTCs	Prognostic association	Wankhede 2022 [33]	CTCs (baseline)	OS HR: 3.03 (95% CI: 2.32–3.98)	Baseline prognostic stratification before or around treatment	CTC positivity was associated with worse OS/DFS, but the evidence in this umbrella review came from a single eligible meta-analysis.

Biomarker class	Evidence domain	Source	Biomarker/analysis	Reported Estimate	Potential clinical decision context	Interpretation/gap
CTCs	Prognostic association	Wankhede 2022 [33]	CTCs (baseline)	DFS HR: 2.95 (95% CI: 1.90–4.59)	Baseline prognostic stratification before or around treatment	CTC positivity was associated with worse OS/DFS, but the evidence in this umbrella review came from a single eligible meta-analysis.
CTCs	Prognostic association	Wankhede 2022 [33]	CTCs (postoperative)	OS HR: 2.80 (95% CI: 1.95–4.02)	Postoperative recurrence-risk stratification	CTC positivity was associated with worse OS/DFS, but the evidence in this umbrella review came from a single eligible meta-analysis.
CTCs	Prognostic association	Wankhede 2022 [33]	CTCs (postoperative)	DFS HR: 2.73 (95% CI: 1.94–3.85)	Postoperative recurrence-risk stratification	CTC positivity was associated with worse OS/DFS, but the evidence in this umbrella review came from a single eligible meta-analysis.

Biomarker class	Evidence domain	Source	Biomarker/analysis	Reported Estimate	Potential clinical decision context	Interpretation/gap
CTCs	Prognostic association	Wankhede 2022 [33]	CTCs (overall)	OS HR: 2.95 (95% CI: 2.37–3.66)	General prognostic stratification	CTC positivity was associated with worse OS/DFS, but the evidence in this umbrella review came from a single eligible meta-analysis.
CTCs	Prognostic association	Wankhede 2022 [33]	CTCs (overall)	DFS HR: 2.97 (95% CI: 2.08–4.22)	General prognostic stratification	CTC positivity was associated with worse OS/DFS, but the evidence in this umbrella review came from a single eligible meta-analysis.
Cytokines and soluble immune biomarkers	Prognostic association	Scirocchi 2022 [34]	sPD-L1, NSCLC-specific	OS HR: 1.81 (95% CI: 1.09–3.00)	Immune-related survival risk stratification in NSCLC	Elevated sPD-L1 was associated with worse OS/PFS, but assay methods and cut-off definitions varied.
Cytokines and soluble immune biomarkers	Prognostic association	Cui 2023 [35]	sPD-L1, NSCLC-specific	OS HR: 2.32 (95% CI: 1.68–3.18)	Immune-related survival risk stratification in NSCLC	Elevated sPD-L1 was associated with worse OS/PFS, but assay methods and cut-off definitions varied.

Biomarker class	Evidence domain	Source	Biomarker/analysis	Reported Estimate	Potential clinical decision context	Interpretation/gap
Cytokines and soluble immune biomarkers	Prognostic association	Scirocchi 2022 [34]	sPD-L1, NSCLC-specific	PFS HR: 2.18 (95% CI: 1.27–3.76)	Immune-related survival risk stratification in NSCLC	Elevated sPD-L1 was associated with worse OS/PFS, but assay methods and cut-off definitions varied.
Cytokines and soluble immune biomarkers	Prognostic association	Cui 2023 [35]	sPD-L1, NSCLC-specific	PFS HR: 2.52 (95% CI: 1.72–3.68)	Immune-related survival risk stratification in NSCLC	Elevated sPD-L1 was associated with worse OS/PFS, but assay methods and cut-off definitions varied.
Cytokines and soluble immune biomarkers	Prognostic association	Cui 2023 [35]	exoPD-L1, exploratory	PFS HR: 4.44 (95% CI: 2.90–6.80)	Immune-checkpoint-related PFS risk stratification	Elevated exoPD-L1 was associated with worse PFS, but evidence remains exploratory and requires validation.
Cytokines and soluble immune biomarkers	Dynamic monitoring	Cui 2023 [35]	exoPD-L1 dynamic change, exploratory analysis	PFS HR: 0.20 (95% CI: 0.06–0.64)	Monitoring treatment-related immune biomarker changes	Decreasing exoPD-L1 was associated with improved PFS, suggesting monitoring potential, but evidence is exploratory.
Cytokines and soluble immune biomarkers	Prognostic association	Liao 2014 [36]	CRP, NSCLC (Inflammation)	OS HR: 1.30 (95% CI: 1.09–1.54)	Inflammation-related survival risk stratification	Elevated CRP was associated with worse OS, but it is a non-specific inflammatory marker.

Biomarker class	Evidence domain	Source	Biomarker/analysis	Reported Estimate	Potential clinical decision context	Interpretation/gap
Cytokines and soluble immune biomarkers	Prognostic association	Liao 2014 [36]	IL-6, NSCLC (Inflammation)	OS HR: 1.80 (95% CI: 1.32–2.46)	Inflammation-related survival risk stratification	Elevated IL-6 was associated with worse OS and may reflect systemic tumor-promoting inflammation.
Cytokines and soluble immune biomarkers	Prognostic association	Liao 2014 [36]	IL-8, NSCLC (Inflammation)	OS HR: 1.93 (95% CI: 0.98–3.81)	Inflammation-related survival risk stratification	Association with OS was not statistically significant based on the reported CI crossing 1.
Cytokines and soluble immune biomarkers	Prognostic association	Liao 2014 [36]	IL-10, NSCLC (Inflammation)	OS HR: 1.62 (95% CI: 0.73–3.57)	Inflammation-related survival risk stratification	Association with OS was not statistically significant based on the reported CI crossing 1.

Note: Reported estimates were extracted from published systematic reviews and meta-analyses and include sensitivity, specificity, area under the receiver operating characteristic curve (AUC), and hazard ratios (HRs) for overall survival (OS), progression-free survival (PFS), recurrence-free survival (RFS), and disease-free survival (DFS), where available. Each row represents one biomarker–outcome pair. Estimates are grouped by biomarker class and evidence domain. No additional statistical pooling was performed in the present umbrella review.

References

20. Page, M.J.; McKenzie, J.E.; Bossuyt, P.M.; Boutron, I.; Hoffmann, T.C.; Mulrow, C.D.; Shamseer, L.; Tetzlaff, J.M.; Akl, E.A.; Brennan, S.E.; et al. The PRISMA 2020 statement: An updated guideline for reporting systematic reviews. *Br. Med. J.* **2021**, *372*, n71.
21. Shea, B.J.; Reeves, B.C.; Wells, G.; Thuku, M.; Hamel, C.; Moran, J.; Moher, D.; Tugwell, P.; Welch, V.; Kristjansson, E.; et al. AMSTAR 2: A Critical Appraisal Tool for Systematic Reviews That Include Randomised or non-randomised Studies of Healthcare interventions, or Both. *BMJ* **2017**, *358*, j4008.
22. Tao, S.; Ju, X.; Zhou, H.; Zeng, Q. Circulating microRNA-145 as a diagnostic biomarker for non-small-cell lung cancer: A systemic review and meta-analysis. *Int. J. Biol. Markers* **2020**, *35*, 51–60.
23. Li, C.; Sun, L.; Zhou, H.; Yang, Y.; Wang, Y.; She, M.; Chen, J.B. Diagnostic value of microRNA-25 in patients with non-small cell lung cancer in Chinese population. *Medicine* **2020**, *99*, e23425–5.
24. Yu, W.; Liu, R.; Miao, Z.; Zhang, L.; Sheyhidin, I.; Ainiwaer, J. The diagnostic significance of blood-derived circRNAs in NSCLC: Systematic review and meta-analysis. *Front. Oncol.* **2022**, *12*, 987704.
25. Pop-Bica, C.; Pintea, S.; Lorand Magdo Cojocneanu, R.; Gulei, D.; Ferracin, M.; Berindan-Neagoe, I. The Clinical Utility of miR-21 and let-7 in Non-small Cell Lung Cancer (NSCLC). A Systematic Review and Meta-Analysis. *Front. Oncol.* **2020**, *10*, 516850.
26. Yuan, Y.; Xu, X.-Y.; Zheng, H.-G.; Hua, B.-J. Elevated miR-21 is associated with poor prognosis in non-small cell lung cancer: A systematic review and meta-analysis. *Eur. Rev. Med. Pharmacol. Sci.* **2018**, *22*, 4166–4180.
27. Xiong, Y.; Wang, T.; Wang, M.; Zhao, J.; Li, X.; Zhang, Z.; Zhou, Y.; Liu, J.; Jia, L.; Han, Y. Long non-coding RNAs function as novel predictors and targets of non-small cell lung cancer: A systematic review and meta-analysis. *Oncotarget* **2018**, *9*, 11377–11386.
28. Chen, C.; Douglas, M.P.; Ragavan, M.V.; Phillips, K.A.; Jansen, J.P. Clinical Validity and Utility of Circulating Tumor DNA (ctDNA) Testing in Advanced Non-small Cell Lung Cancer (aNSCLC): A Systematic Literature Review and Meta-analysis. *Mol. Diagn. Ther.* **2024**, *28*, 525–536.
29. Guo, R.Q.; Peng, J.Z.; Sun, J.; Li, Y.M. Clinical significance of circulating tumor DNA in localized non-small cell lung cancer: A systematic review and meta-analysis. *Clin. Exp. Med.* **2022**, *23*, 1621–1631.
30. Maffeo, D.; Rina, A.; Serio, V.B.; Markou, A.; Powrózek, T.; Constâncio, V.; Nunes, S.P.; Jerónimo, C.; Calvo, A.; Mari, F.; et al. The Evidence Base for Circulating Tumor DNA-Methylation in Non-Small Cell Lung Cancer: A Systematic Review and Meta-Analysis. *Cancers* **2024**, *16*, 3641.
31. Lu, J.; Feng, Y.; Guo, K.; Sun, L.; Ruan, S.; Zhang, K. Prognostic value of preoperative circulating tumor DNA in non-small cell lung cancer: A systematic review and meta-analysis. *J. Cancer Res. Clin. Oncol.* **2024**, *150*, 25.
32. Sun, X.; Abrahamson, P.; Ballew, N.; Kalilani, L.; Phiri, K.; Bell, K.F.; Slowley, A.; Zajac, M.; Hofstatter, E.; Stojadinovic, A.; et al. The Utility of ctDNA in Lung Cancer Clinical Research and Practice: A Systematic Review and Meta-Analysis of Clinical Studies. *Cancer Investig.* **2023**, *41*, 571–592.
33. Durgesh Wankhede Grover, S.; Hofman, P. Circulating Tumor Cells as a Predictive Biomarker in Resectable Lung Cancer: A Systematic Review and Meta-Analysis. *Cancers* **2022**, *14*, 6112.
34. Scirocchi, F.; Strigari, L.; Filippo, A.D.; Napoletano, C.; Pace, A.; Rahimi, H.; Botticelli, A.; Rughetti, A.; Nuti, M.; Zizzari, I.G. Soluble PD-L1 as a Prognostic Factor for Immunotherapy Treatment in Solid Tumors: Systematic Review and Meta-Analysis. *Int. J. Mol. Sci.* **2022**, *23*, 14496–6.
35. Cui, Q.; Li, W.; Wang, D.; Wang, S.; Yu, J. Prognostic significance of blood-based PD-L1 analysis in patients with non-small cell lung cancer undergoing immune checkpoint inhibitor therapy: A systematic review and meta-analysis. *World J. Surg. Oncol.* **2023**, *21*, 318.
36. Liao, C.; Yu, Z.; Guo, W.; Liu, Q.; Wu, Y.; Li, Y.; Bai, L. Prognostic value of circulating inflammatory factors in non-small cell lung cancer: A systematic review and meta-analysis. *Cancer Biomark.* **2014**, *14*, 469–481.
