# Supplementary material for: Diagnostic and Prognostic Roles of Blood-Based Immune Biomarkers in Non-Small Cell Lung Cancer: An Umbrella Review of Systematic Reviews and Meta-Analyses
Source: Life (Basel). 2026 Jul 7;16(7):1130. doi: 10.3390/life16071130 (PMC13412772; doi:10.3390/life16071130)
Supplement: Supplementary file 1 [file life-16-01130-s001.zip › Zhao_Ling_Li_Kam_Supplementary Table 1_final_May_27.pdf]

PRISMA 2020 Checklist

Section and Topic	Item #	Checklist item	Location where item is reported
**TITLE**			
Title	1	Identify the report as a systematic review.	Title page: title identifies the article as an “Umbrella Review of Systematic Reviews and Meta-Analyses”.
**ABSTRACT**			
Abstract	2	See the PRISMA 2020 for Abstracts checklist.	Abstract: background, methods, results, and conclusions are reported in the structured abstract.
**INTRODUCTION**			
Rationale	3	Describe the rationale for the review in the context of existing knowledge.	Introduction, paragraphs 1–3: rationale for blood-based immune biomarkers and fragmented evidence in NSCLC.
Objectives	4	Provide an explicit statement of the objective(s) or question(s) the review addresses.	Introduction, final paragraph: objective to systematically review 57 NSCLC-related reviews and summarize diagnostic, prognostic, and monitoring value.
**METHODS**			
Eligibility criteria	5	Specify the inclusion and exclusion criteria for the review and how studies were grouped for the syntheses.	Methods, paragraphs 1–2: eligibility criteria, biomarker categories, blood-based specimens, and grouping by biomarker class.
Information sources	6	Specify all databases, registers, websites, organisations, reference lists and other sources searched or consulted to identify studies. Specify the date when each source was last searched or consulted.	Methods, paragraph 2: PubMed searched on 25 May 2025; Systematic

Section and Topic	Item #	Checklist item	Location where item is reported
			Review and Meta-Analysis filters applied.
Search strategy	7	Present the full search strategies for all databases, registers and websites, including any filters and limits used.	Methods, paragraph 2: PubMed search terms reported as ((NSCLC) OR (non-small cell lung cancer)) AND (biomarkers), with filters and limits described.
Selection process	8	Specify the methods used to decide whether a study met the inclusion criteria of the review, including how many reviewers screened each record and each report retrieved, whether they worked independently, and if applicable, details of automation tools used in the process.	Methods, paragraph 3: Covidence screening process; three reviewers screened records and full texts; disagreements resolved by discussion.
Data collection process	9	Specify the methods used to collect data from reports, including how many reviewers collected data from each report, whether they worked independently, any processes for obtaining or confirming data from study investigators, and if applicable, details of automation tools used in the process.	Methods, paragraph 4: data extraction focused on pooled effect estimates from included systematic reviews/meta-analyses. Reviewer-level details are described in Authorship contributions.
Data items	10a	List and define all outcomes for which data were sought. Specify whether all results that were compatible with each outcome domain in each study were sought (e.g. for all measures, time points, analyses), and if not, the methods used to decide which results to collect.	Methods, paragraphs 4 and 6: outcomes included HRs for OS, PFS, RFS, DFS and diagnostic measures including sensitivity, specificity, and AUC.
	10b	List and define all other variables for which data were sought (e.g. participant and intervention characteristics, funding sources). Describe any assumptions made about any missing or unclear information.	Methods, paragraphs 1 and 4:

Section and Topic	Item #	Checklist item	Location where item is reported
			variables included biomarker type/class, endpoint, pooled effect estimate, and source review/meta-analysis. No assumptions about missing or unclear information were reported.
Study risk of bias assessment	11	Specify the methods used to assess risk of bias in the included studies, including details of the tool(s) used, how many reviewers assessed each study and whether they worked independently, and if applicable, details of automation tools used in the process.	Methods, paragraph 5: AMSTAR 2 used; two reviewers independently assessed included reviews and resolved disagreements by consensus.
Effect measures	12	Specify for each outcome the effect measure(s) (e.g. risk ratio, mean difference) used in the synthesis or presentation of results.	Methods, paragraph 6: HRs for time-to-event outcomes; sensitivity, specificity, and AUC for diagnostic biomarkers.
Synthesis methods	13a	Describe the processes used to decide which studies were eligible for each synthesis (e.g. tabulating the study intervention characteristics and comparing against the planned groups for each synthesis (item #5)).	Methods, paragraphs 1, 4, and 6: studies grouped by biomarker category and clinical endpoint for descriptive synthesis.
	13b	Describe any methods required to prepare the data for presentation or synthesis, such as handling of missing summary statistics, or data conversions.	Methods, paragraphs 4 and 6: published pooled estimates were extracted and compared descriptively; no individual patient-level data were reanalysed and no additional re-pooling

Section and Topic	Item #	Checklist item	Location where item is reported
			was performed.
	13c	Describe any methods used to tabulate or visually display results of individual studies and syntheses.	Methods, paragraph 7; Results Tables 1–4 and Figures 2–5: tabular and visual display of published pooled estimates.
	13d	Describe any methods used to synthesize results and provide a rationale for the choice(s). If meta-analysis was performed, describe the model(s), method(s) to identify the presence and extent of statistical heterogeneity, and software package(s) used.	Methods, paragraphs 4 and 6: descriptive and visual synthesis only; no new meta-analysis or statistical re-pooling was performed because of heterogeneity and likely primary-study overlap.
	13e	Describe any methods used to explore possible causes of heterogeneity among study results (e.g. subgroup analysis, meta-regression).	Not applicable: no new meta-analysis, subgroup analysis, or meta-regression was performed. Heterogeneity is discussed descriptively in Methods and Limitations.
	13f	Describe any sensitivity analyses conducted to assess robustness of the synthesized results.	Not applicable: no new statistical synthesis was performed; therefore no sensitivity analyses were conducted.
Reporting bias assessment	14	Describe any methods used to assess risk of bias due to missing results in a synthesis (arising from reporting biases).	Not applicable/not assessed in this umbrella review because no new statistical synthesis or re-pooling was performed. Limitations discuss possible missing

PRISMA 2020 Checklist

Section and Topic	Item #	Checklist item	Location where item is reported
			evidence due to PubMed-only search.
Certainty assessment	15	Describe any methods used to assess certainty (or confidence) in the body of evidence for an outcome.	Methods, paragraph 5 and Results opening paragraph: methodological confidence assessed using AMSTAR 2.
**RESULTS**			
Study selection	16a	Describe the results of the search and selection process, from the number of records identified in the search to the number of studies included in the review, ideally using a flow diagram.	Methods, paragraph 3; Figure 1 PRISMA flow diagram and caption: numbers identified, screened, excluded, assessed, and included.
	16b	Cite studies that might appear to meet the inclusion criteria, but which were excluded, and explain why they were excluded.	Figure 1 caption: full-text exclusion reasons summarized. Individual excluded studies are not cited in the manuscript.
Study characteristics	17	Cite each included study and present its characteristics.	Results sections and Tables 1–4: included source reviews cited and their extracted biomarker/outcome characteristics presented.
Risk of bias in studies	18	Present assessments of risk of bias for each included study.	Results opening paragraph: AMSTAR 2 overall ratings summarized.
Results of individual studies	19	For all outcomes, present, for each study: (a) summary statistics for each group (where appropriate) and (b) an effect estimate and its precision (e.g. confidence/credible interval), ideally using structured tables or plots.	Results sections; Tables 1–4; Figures 2–5: effect estimates and confidence intervals/diagnostic values for each included review-

Section and Topic	Item #	Checklist item	Location where item is reported
			level result.
Results of syntheses	20a	For each synthesis, briefly summarise the characteristics and risk of bias among contributing studies.	Results opening paragraph: AMSTAR 2 quality distribution summarized; Results biomarker sections summarize characteristics of contributing reviews.
	20b	Present results of all statistical syntheses conducted. If meta-analysis was done, present for each the summary estimate and its precision (e.g. confidence/credible interval) and measures of statistical heterogeneity. If comparing groups, describe the direction of the effect.	Results sections; Tables 1–4; Figures 2–5: published pooled estimates and confidence intervals/diagnostic values presented. No new meta-analysis was conducted.
	20c	Present results of all investigations of possible causes of heterogeneity among study results.	Not applicable: no subgroup analysis, meta-regression, or formal investigation of heterogeneity was conducted in this umbrella review.
	20d	Present results of all sensitivity analyses conducted to assess the robustness of the synthesized results.	Not applicable: no sensitivity analyses were conducted because no new pooled estimates were generated.
Reporting biases	21	Present assessments of risk of bias due to missing results (arising from reporting biases) for each synthesis assessed.	Not applicable/not assessed: reporting bias due to missing results was not formally assessed for each synthesis in this umbrella review.
Certainty of evidence	22	Present assessments of certainty (or confidence) in the body of evidence for each outcome assessed.	Results opening paragraph and Limitations: AMSTAR 2 overall confidence ratings

Section and Topic	Item #	Checklist item	Location where item is reported
			reported and used to interpret the evidence base.
**DISCUSSION**			
Discussion	23a	Provide a general interpretation of the results in the context of other evidence.	Discussion, paragraphs 1–5: interpretation of results across biomarker classes in the context of other evidence.
	23b	Discuss any limitations of the evidence included in the review.	Limitations: limitations of included evidence, including heterogeneity, small numbers for some biomarker classes, overlap of primary studies, and low/critically low AMSTAR 2 ratings.
	23c	Discuss any limitations of the review processes used.	Limitations: limitations of review process, including PubMed-only search, reliance on published reviews/meta-analyses, and inability to harmonize endpoints or re-pool data.
	23d	Discuss implications of the results for practice, policy, and future research.	Discussion final paragraph and Conclusion: implications for clinical interpretation, assay standardization, prospective validation, and future multimodal comparative

PRISMA 2020 Checklist

Section and Topic	Item #	Checklist item	Location where item is reported
			studies.
**OTHER INFORMATION**			
Registration and protocol	24a	Provide registration information for the review, including register name and registration number, or state that the review was not registered.	Methods, paragraph 1: PROSPERO registration CRD420261350843.
	24b	Indicate where the review protocol can be accessed, or state that a protocol was not prepared.	Methods, paragraph 1: protocol registered in PROSPERO. Access location is PROSPERO registration record CRD420261350843.
	24c	Describe and explain any amendments to information provided at registration or in the protocol.	Not reported: no amendments to the registered protocol are described in the manuscript.
Support	25	Describe sources of financial or non-financial support for the review, and the role of the funders or sponsors in the review.	Funding statement: Jiangsu Provincial University Key Laboratory for Smart Diagnosis and Treatment of Lung Cancer (Project Code: 26KINFD011) and Duke Kunshan University Summer Research Scholars Program. Role of funders not specified.
Competing interests	26	Declare any competing interests of review authors.	Conflict of interest statement: authors declare no conflicts of interest.
Availability of data, code and other materials	27	Report which of the following are publicly available and where they can be found: template data collection forms; data extracted from included studies; data used for all analyses; analytic code; any other materials used in the review.	Data availability statement: extracted review-level data and materials available from the corresponding author upon

PRISMA 2020 Checklist

Section and Topic	Item #	Checklist item	Location where item is reported
			reasonable request.

From: Page MJ, McKenzie JE, Bossuyt PM, Boutron I, Hoffmann TC, Mulrow CD, et al. The PRISMA 2020 statement: an updated guideline for reporting systematic reviews. BMJ 2021;372:n71. doi: 10.1136/bmj.n71. This work is licensed under CC BY 4.0. To view a copy of this license, visit https://creativecommons.org/licenses/by/4.0/
